# Ultrasound Examination Facilitated the Diagnosis of an Intercostal Schwannoma

**DOI:** 10.7759/cureus.26079

**Published:** 2022-06-19

**Authors:** Wei-Ting Wu, Ke-Vin Chang, Levent Ozcakar

**Affiliations:** 1 Physical Medicine and Rehabilitation, National Taiwan University Hospital Bei-Hu Branch, Taipei, TWN; 2 Physical Medicine and Rehabilitation, Hacettepe University Medical School, Ankara, TUR

**Keywords:** schwannoma, intercostal, neurilemmoma, thorax, ultrasonography

## Abstract

Ultrasound imaging is known for its capability in scrutinizing superficial soft tissue disorders. We report a rare case of a 71-year-old male who presented with a history of rectal cancer with lymph node metastasis and had complete remission after surgery and chemotherapy. He had a palpable mass over the right anterior lower chest, which became gradually painful in the recent six months. Ultrasound facilitated the diagnosis of an intercostal schwannoma, which was also evident on magnetic resonance imaging and was confirmed by the histopathological study. Following the tumor excision, the patient had complete pain relief at the second-month follow-up. The case report highlighted the usefulness of ultrasound in clarification of the exact location of a chest wall tumor in relation to ribs, pleura, adjacent muscles, and intercostal neurovascular bundles as well as delineation of its echotexture and internal vascularity.

## Introduction

Based on its excellent resolution regarding superficial structures, high-frequency ultrasound (US) has been widely employed for imaging soft tissue pathologies [1]. Compared with computed tomography and magnetic resonance imaging, it has benefits like better accessibility, portability, free of radiation, and allowance of dynamic examination [1]. For a chest wall lesion, US can be applied for clarification of the exact location in relation to ribs, pleura, adjacent muscles, and intercostal neurovascular bundles [2]. It can easily assess the echotexture and internal vascularity as well [3]. Herein, we report a rare case of a 71-year-old male with a palpable mass over the right anterior lateral lower chest whereby US facilitated the diagnosis of an intercostal schwannoma.

## Case presentation

A 71-year-old male had been diagnosed with rectal cancer with lymph node metastasis 12 years ago. He had undergone a laparoscopic low anterior resection and adjuvant chemotherapy with complete remission thereafter. One year ago, he accidentally found a palpable mass over the right anterior lateral lower chest. Initially, the mass had not been painful; however, in the last three months, he started to experience intermittent shooting pain without any enlargement of the mass. Recently, he visited the surgical department for consultation and was then referred for US examination.

**Figure 1:**
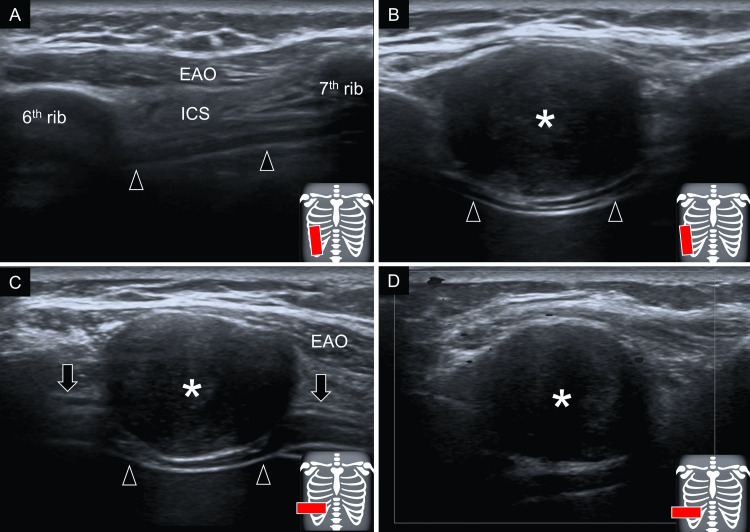
Ultrasound imaging of the intercostal space of the right lower ribs. The transducer is first placed over the right anterior lateral lower chest wall in the sagittal plane (A) and a mass (asterisks) emerged from the sixth intercostal space when relocating the transducer more laterally (B). The transducer was then pivoted to the horizontal plane to see the intercostal nerve (arrows) connecting to the mass (C). The power Doppler imaging showed a petechial increase of vascularity (D). (Arrowheads showing pleura and red square showing the footprint of the transducer.) EAO: external abdominal oblique muscle; ICS: intercostal muscle

The transducer was first placed in the sagittal plane along the right anterior axillary line to visualize the intercostal space of the lower ribs (Figure 1A). Then it was gradually relocated toward the mid-axillary line. A circular, solid, and hypoechoic mass was seen emerging from the inferior border of the sixth rib - also protruding toward the overlying external abdominal oblique muscle (Figure 1B). Its inferior border was visualized compressing the intercostal innermost muscle and pleura. The transducer was pivoted in the horizontal plane where two hypoechoic tubular structures were found stemming from the medial and lateral edges of the tumor (Figure 1C). The mass also displayed mild power Doppler activity (Figure 1D).

**Figure 2:**
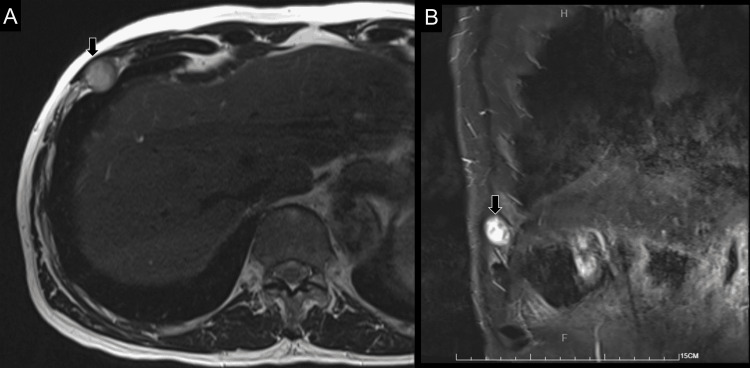
T2-weighted MRI of the intercostal tumor (arrows) in the axial (A) and coronal (B) views.

Magnetic resonance imaging revealed a well-circumscribed nodular tumor with a diameter of 2 cm and heterogeneous high signals on T2-weighted imaging at the right sixth intercostal space (Figure 2).

An indentation to the adjacent pleura was also evident. As the lesion could have been a metastatic tumor, surgical excision was scheduled. Histological examination yielded spindle cells with wavy nuclei in fibromyxoid stroma, positive for S100 protein and negative for epithelial membrane antigen and smooth muscle actin [4]. Confirming the diagnosis of a schwannoma, the patient was called for follow-up with complete pain relief on the second post-operative month.

## Discussion

Ultrasound (US) imaging excels in delineating superficial soft tissue disorders. In the lower chest region, the most common palpable lesion is lipoma, a benign tumor of non-cancerous growth of adipocytes [5]. Most subcutaneous lipomas are rarely painful unless the adjacent cutaneous nerves are compressed [6]. Relevant US findings include an ovoid or elliptical shape with striated echogenic lines [7]. Another prevalent superficial tumor is the epidermoid cyst. It is derived from ectodermal tissues and is filled with keratin [8]. Upon US examination, it appears as oval, homogenous, hypoechoic, and well-demarcated [7]. A punctum extending to the skin may be recognized at its superficial portion, where posterior enhancement can be seen at its deepest border. Doppler US images occasionally disclose increased vascularity at the peripheral portion of the cyst when it is ruptured, inflamed, or infected. In our case, the mass was located underneath the external abdominal oblique muscle, making the two aforementioned pathologies less likely.

The location of the present tumor might be the hint indicative of a peripheral nerve sheath tumor. US examination easily identified it occupying the intercostal space and protruding to adjacent muscles and underlying pleura. As the tumor had clear margins, it was less likely to have originated from the muscles or lungs. Further, despite the patient's history of rectal cancer, his homogenous echotexture and mild vascularity were not consistent with a metastatic tumor [9]. Therefore, we considered that the mass could have possibly originated from intercostal neurovascular structures. Taking into account the sonographic features of vascular-derived tumors, i.e., prominent Doppler activity and multiple tubular architecture, schwannoma seemed to overweigh the differential diagnoses [10].

The second hint for the present tumor lied in the horizontal imaging. Two tubular hypoechoic structures were seen connecting to the medial and lateral edges of the tumor. It is well known that the high-resolution US provides a clear depiction of the peripheral nerves which appear as hypoechoic and fibrillar in their long axes. Likewise, US imaging was suggestive of a mass emerging from the intercostal nerve. US findings of a schwannoma might be variable, i.e., homogenous or heterogeneous as well as hypoechoic or hyperechoic [11-13]. In large schwannomas, intratumor hemorrhage, fibrosis, calcification, and cyst formation are not rare. The presence of “rat tail” appearance would also be suggestive [14]. In contrast to neurofibromas, it is also common to have internal vascularity on Doppler imaging. The eccentric location of the schwannoma in relation to the accompanying nerve is another characteristic finding in comparison to neurofibromas which are commonly concentrically located [15].

The intercostal schwannoma is an uncommon tumor of the chest wall. It is usually painless but may incur intermittent pain in some cases [16]. It is slow growing and its prevalence is similar across both sexes. As it is well-encapsulated, surgical excision mostly yields satisfactory outcomes without sequelae, e.g., sensory deficit or persistent intercostal neuralgia. Although US imaging is proven useful in the detection of an intercostal schwannoma, subsequent contrast-enhanced computed tomography or magnetic resonance imaging is still mandatory to examine possible invasions to the ribs and lung.

## Conclusions

In patients with a palpable chest wall tumor, the intercostal schwannoma should be taken into the possible diagnosis. US imaging is helpful for clarifying its location, which typically occupies the intercostal space and may protrude toward the adjacent muscles and pleura. The view in the horizontal plane facilities the delineation of its eccentric localization in relation to the intercostal nerve. Subsequent contrast-enhanced computed tomography or magnetic resonance imaging is still required considering its possible invasion to the ribs and lungs.
